# Contributions of Artificial Intelligence to Decision Making in Nursing: A Scoping Review Protocol

**DOI:** 10.3390/nursrep13010007

**Published:** 2023-01-06

**Authors:** Filipe Fernandes, Paulo Santos, Luís Sá, José Neves

**Affiliations:** 1 Institute of Health Sciences, Center for Interdisciplinary Research in Health (CIIS), Escola Superior de Saúde do Vale do Ave—Instituto Politécnico de Saúde do Norte, Vila Nova de Famalicão, Universidade Católica Portuguesa, 4169-005 Porto, Portugal; 2 Institute of Health Sciences, Center for Interdisciplinary Health Research (CIIS), Escola Superior de Saúde da Cruz Vermelha Portuguesa, 1300-125 Lisboa, Portugal; 3 Institute of Health Sciences, Center for Interdisciplinary Research in Health (CIIS), Universidade Católica Portuguesa, 4169-005 Porto, Portugal; 4 Research Unit on Artificial Intelligence & Health, Algoritmi Centre, University of Minho, 4710-057 Braga, Portugal

**Keywords:** nursing, artificial intelligence, decision making, protocol review

## Abstract

Background: Artificial intelligence (AI) techniques and methodologies for problem solving are emerging as formal tools essential to assist in nursing care. Given their potential to improve workflows and to guide decision making, several studies have been developed; however, little is known about their impact, particularly on decision making. Objective: The aim of this study was to map the existing research on the use of AI in decision making in nursing. With this review protocol, we aimed to map the existing research on the use of AI in nursing decision making. Methods: A scoping review was conducted following the framework proposed by the Joanna Briggs Institute (JBI). The search strategy was tailored to each database/repository to identify relevant studies. The contained articles were the targets of the data extraction, which was conducted by two independent researchers. In the event of discrepancies, a third researcher was consulted. Results: This review included quantitative, qualitative and mixed method studies. Primary studies, systematic reviews, dissertations, opinion texts and gray literature were considered according to the three steps that the JBI has defined for scoping reviews. Conclusions: This scoping review synthesized knowledge that could help advance new scientific developments and find significant and valuable outcomes for patients, caregivers and leaders in decision making. This review was also intended to encourage the development of research lines that may be useful for the development of AI tools for decision making.

## 1. Introduction

Nursing as a discipline, but also as a health profession, has sought to respond to new challenges and to develop its own knowledge to endow its professionals with more and better skills to provide quality nursing care based o n the best scientific knowledge available. Nurses are required to have greater responsibility and autonomy in decision making, since this same competence is reflected in the regulation of the professional practice [1,2].

In recent decades, cognitive sciences, social and cognitive psychology and neurosciences have made efforts to understand how conscious and unconscious mechanisms interact, influencing our behavior and decision making [3].

The multidimensionality of the human being has led several authors to study the mechanisms and the role of the conscious and the unconscious in the decision-making process. Study approaches have been diverse, highlighting the importance attributed to neuronal structures by different authors over the years [4,5,6]

With regard to nursing, this is an essential aspect with great emphasis both in discipline and in clinical practice in helping managers, namely when we realize that nursing integrates professionals who are, above all, humans beings with complex problems which can influence the decision making process. This process is highly complex as it simultaneously requires knowledge, experience, critical thinking and intuition and, concomitantly, creativity, empathy and compassion in addition to other aspects, such as the emotional and relational nature [1,7,8].

Internationally, studies address the domain of clinical decisions in nursing, with many countries trying to follow this dynamic [9,10]. However, many aspects of the subject need further investigation, namely with regard to knowledge of the aspects that influence this clinical decision making process [11,12,13]

In this way, it is important that caregivers and health organizations integrate systems and tools resulting from technological advances to improve the efficiency and quality of health care. Artificial intelligence (AI) is an example of this, with the potential to improve the quality of nursing care by increasing the efficiency and patient safety at a low cost [14].

According to Markets and Markets, the “AI in healthcare market is projected to grow from USD 6.9 billion in 2021 to reach USD 67.4 billion by 2027” [15]. Inherently, this investment will have an impact on nursing care, and it is important that nurses acquire knowledge about the new tools and their implications in their daily professional life.

AI has revolutionized the way we think about the world, has contributed to countless advances in healthcare and has enormous potential to improve the quality of care. These tools aim to develop computer systems that mimic human thinking [16] to, in the case of this study, anticipate, predict and facilitate the decision-making process [17,18]. Overall, AI can aid nurses, not substitute them, in decision making and will help them make more self-assured decisions. The fear and distrust that may exist regarding the use of new AI technologies can only be overcome with knowledge and an understanding of the existing evidence. Mapping existing evidence about the contributions of AI to decision making in nursing will help clarify these aspects. Additionally, despite the recognition of the importance of AI tools in health care, there is a dispersion of knowledge in the reference literature that is important to synthesize, favoring its transfer to practice.

## 2. Methods

In order to find literature reviews on this topic, a preliminary search was initially carried out in the CINAHL Complete, MEDLINE Complete and Cochrane Database of Systematic Reviews databases via EBSCOhost, and no similar reviews were found on this topic. Thus, the authors considered it pertinent to carry out a scoping review based on the methodology proposed by the Joanna Briggs Institute [19] Once the dispersed existing knowledge was examined and mapped with this review, it became the starting point for further research.

The scoping review methodology aimed to map the amount of knowledge in a given knowledge area, so we considered this type of review appropriate given the emerging nature of the topic [20]. Therefore, we intended to map the studies undertaken within the framework of AI’s contributions to nursing decision making and provided a comprehensive overview of what already was, how it was and in which contexts the topic was studied to analyze the aspects related to the contributions of AI to the decision making process in nursing care as well as to identify and to analyze gaps in the available knowledge on the subject with the aim of developing a research project.

This review protocol was registered in the Open Science Framework (OSF) platform, and the results were reported according to the guidelines of the Preferred Reporting Items for Systematic and Meta-Analyses extension for Scoping Reviews ((PRISMA-ScR) [21].

### 2.1. Research Question

Based on the JBI recommendations contained in the PPC mnemonics [20], the following question was elaborated: What evidence has been published regarding the contributions of AI to decision making in nursing? For this scoping review, the selected inclusion criteria are as follows:

Participants—this review considered all studies involving nurses working in healthcare sector, regardless of age, gender, employment status or work experience;

Concept—this review considered all studies addressing the use of AI in decision making in nursing;

Context—this review considered studies related to hospital care where AI was used, regardless of the public or private nature of the institution.

In addition to the central question of this study, we wanted to answer the following secondary questions:

What methods have been used to study the contribution of AI to decision making in nursing?

In what contexts has the contribution of AI been studied in decision making in nursing?

What factors were identified in the study of the contribution of AI to decision making in nursing?

### 2.2. Search Strategy

This review included quantitative, qualitative and mixed method studies. Primary studies, systematic reviews, dissertations, opinion pieces and gray literature were considered according to the three steps defined by the JBI for scoping reviews [20].

Only open access documents in Portuguese, English and Spanish were considered with no time or geographic limitations, aiming to consider how AI is studied and perceived in contexts with which we culturally identify. As long as they met the eligibility criteria, authors may have included other documents that they deemed relevant to this review.

The outlined search strategy aimed to find published and unpublished studies. For this purpose, an electronic search was conducted in the CINAHL Complete; MEDLINE Complete; Nursing & Allied Health Collection: Comprehensive; Cochrane Central Register of Controlled Trials; Cochrane Database of Systematic Reviews; Cochrane Methodology Register; Library, Information Science & Technology Abstracts; MedicLatina (via EBSCO); SciELO; Scopus; LILACS; and JBI Database of Systematic Reviews databases. The choice for these resources was justified by the fact that they serve to disseminate knowledge in health sciences, particularly in nursing. We also considered the research in information sources linked to AI, which proved to be inconsequential. To find unpublished studies, we searched the RCAAP, Capes Thesis Portal and OpenGrey databases.

This research was developed in three stages [20]. In the first stage, an initial search of the MEDLINE Complete and CINAHL Complete databases (via EBSCO) was performed to identify the key descriptors and keywords more commonly used in the title and abstract with the MESH descriptors “Artificial Intelligence”, “Nursing” and “Decision-making”.

The second stage was developed by combining keywords and indexed terms in a unique search strategy according to the specifics of each database. In the third stage, the bibliographic references of all selected studies were analyzed in order to include other studies considered to be of interest.

All citations were loaded using Mendeley Desktop software (version 1.19.5, Mendeley Ltd., Elsevier, Amsterdam, The Netherlands). Duplicate citations were removed, and the titles and abstracts of studies were screened following eligibility determined by two independent reviewers.

A third reviewer may have been required to clarify any discrepancies. Documents that met the eligibility criteria were read in their entirety following the text analysis process. Reasons for exclusion of studies after full reading that did not meet the eligibility criteria were recorded and reported in the scoping review.

### 2.3. Data Extraction

Data extraction for the scoping review was carried out by two independent reviewers after confirming the relevance of previously selected publications. For data extraction, a tool developed by the researchers was used (Table 1) according to the specific details of the population, concept and context. It is important to note that this tool was preliminary and may have been subject to adjustments resulting from the reading and analysis of eligible publications during the review process. The researchers conducted a pilot test to familiarize themselves with the tool. In case of need for additional clarification during this process, the authors of the publications under analysis may have been contacted.

### 2.4. Data Synthesis

The evidence found in the literature was presented in a descriptive way which was in line with the scope and objectives of scoping review using tables and/or charts. This process was carried out independently by each of the reviewers involved in the previous step, and a third reviewer may later have been involved to obtain consensus on the differences found.

## 3. Presentation and Interpretation of Results

The data collected was presented in a narrative format, which may have included a table, chart or organization chart in order to map the evidence available in the literature regarding the contributions of AI to decision making in nursing. In this way, it was possible to identify gaps in the available knowledge and to analyze the typology of the studies, the AI tools studied and the contexts and aspects associated with decision making.

The scoping review that we proposed will contribute to the dissemination of knowledge in this area of accelerated development and will have a strong impact throughout the world. By providing valuable and structured information related to the contributions of AI to decision making in nursing, nurses, leaders and managers will be able to implement preventive/corrective measures that promote greater patient safety and a greater satisfaction of nurses.

## 4. Conclusions

In recent years, several studies have been conducted in the field of AI in healthcare. However, little is known about its impact on nursing decision making. The galloping development of these tools applied to health is proving to be both an opportunity and a necessity for nurses looking for a higher efficiency and quality in the delivery of care.

The large-scale adoption of algorithmic learning in nursing care and decision making is not devoid of ethical issues of which we need to be aware to ensure that the construction of such algorithms, as in other processes involving humans, is based on an ethical conscience. It is predictable that the implementation of AI tools will change the way nurses interact with patients and their families. However, the importance of person-centered high-quality compassionate nursing care is not threatened by new technologies, but, rather, it is reinforced.

With this scoping review, we hoped to synthesize knowledge that could help fuel new scientific developments and find significant and valuable outcomes for care recipients, caregivers, leaders and managers in decision making.

This review also sought to encourage the development of research lines that may be useful for the development of these tools for nursing decision making.

## Figures and Tables

**Table 1:** Data extraction tool.

	Responsible for extraction: ______________________
Title	Contributions of artificial intelligence to decision making in nursing: a scoping review protocol
Research Question	What evidence is there about the contribution of AI to decision making in nursing? Secondary Questions: What methodologies have been used to study the contribution of AI in decision making in nursing? In what contexts has the contribution of AI been studied in decision making in nursing? What aspects were identified in the study of the contributions of AI to decision making in nursing?
Eligibility Criteria	Participants—this review considered all studies that included nurses providing care, regardless of age, sex, employment relationship and professional experience; Concept—this review considered all studies that address AI in decision making in nursing; Context—This review considered studies in the context of hospital care in which AI was used, regardless of the public or private nature of the institution.
Characteristics of Source of Evidence	Authors:___________________________________________________ Year of publication:_________________________________________ Geographic location and clinical context:_______________________ Study type and design:______________________________________ Objectives:_________________________________________________ Sample size:________________________________________________ Concepts relevant to the review questions:_____________________
Results Extracted from the Source of Evidence	Study Results: ______________________________________________ Limitations:________________________________________________ Future research recommendations:____________________________ Bibliography cited:__________________________________________ Comments:________________________________________________
